# High Resolution Copy Number Variation Data in the NCI-60 Cancer Cell Lines from Whole Genome Microarrays Accessible through CellMiner

**DOI:** 10.1371/journal.pone.0092047

**Published:** 2014-03-26

**Authors:** Sudhir Varma, Yves Pommier, Margot Sunshine, John N. Weinstein, William C. Reinhold

**Affiliations:** 1 Laboratory of Molecular Pharmacology, Center for Cancer Research, National Cancer Institute, National Institutes of Health, Bethesda, Maryland, United States of America; 2 HiThru Analytics LLC, Laurel, Maryland, United States of America; 3 Systems Research and Applications Corporation, Fairfax, Virginia, United States of America; 4 Departments of Bioinformatics and Computational Biology and Department of Systems Biology, M.D. Anderson Cancer Center, Houston, Texas, United States of America; The Chinese University of Hong Kong, Hong Kong

## Abstract

Array-based comparative genomic hybridization (aCGH) is a powerful technique for detecting gene copy number variation. It is generally considered to be robust and convenient since it measures DNA rather than RNA. In the current study, we combine copy number estimates from four different platforms (Agilent 44 K, NimbleGen 385 K, Affymetrix 500 K and Illumina Human1Mv1_C) to compute a reliable, high-resolution, easy to understand output for the measure of copy number changes in the 60 cancer cells of the NCI-DTP (the NCI-60). We then relate the results to gene expression. We explain how to access that database using our CellMiner web-tool and provide an example of the ease of comparison with transcript expression, whole exome sequencing, microRNA expression and response to 20,000 drugs and other chemical compounds. We then demonstrate how the data can be analyzed integratively with transcript expression data for the whole genome (26,065 genes). Comparison of copy number and expression levels shows an overall medium high correlation (median r = 0.247), with significantly higher correlations (median r = 0.408) for the known tumor suppressor genes. That observation is consistent with the hypothesis that gene loss is an important mechanism for tumor suppressor inactivation. An integrated analysis of concurrent DNA copy number and gene expression change is presented. Limiting attention to focal DNA gains or losses, we identify and reveal novel candidate tumor suppressors with matching alterations in transcript level.

## Introduction

The NCI-60 is a set of 60 widely used cancer cell lines derived from 9 tissues of origin including breast, central nervous system, colon, lung, prostate, ovary and kidney, as well as leukemia and melanomas [1]. We, and others, have previously made available molecular data on multiple platforms for the NCI-60 [2]–[7], making it a unique resource for both pharmacogenomics [8], [9] and systems biology [10], [11]. These cell lines retain gene expression patterns from their original cancer tissues-of-origin, as demonstrated by co-clustering [4], and comparison to clinical samples [12]. The ability to compare drug response and genomic data for these cell lines is unmatched by any other clinical or cancer cell databases [8], [11], [13], [14].

Prior studies of DNA copy number using aCGH from multiple cancerous cell lines and clinical samples have enhanced understanding of DNA variability at the cellular level [15], as well as yielding translational insights [16]. aCGH provides a measurement of genomic instability [17], a hallmark of carcinogenesis [18]. Associations between gene copy number and expression have also been studied, in some cases yielding implications regarding mechanisms of cancer progression [19], [20].

Data on multiple platforms profiling the NCI-60 are accessible through our CellMiner web application [21]. Recently, we have introduced web-based tools that allow the non-bioinformatician to assess and cross-compare the databases [8]. In the current study, we expand this integrative capacity by presenting the high-resolution DNA copy number data for the NCI-60 synthesized from the combination of data from four platforms (Table S1), and placed it in a format stereotypical to the other forms of data. We introduce the “Gene DNA copy number” web-tool, designed to allow the non-bioinformatician, to query, visualize and download relative DNA copy number data. The output from this tool facilitates integration of DNA copy data with our other databases, enhancing their integrative capacity.

Analytically, we provide measurements of relative DNA copy number variation within and between cell lines, compute several measures of genomic instability, and correlate relative DNA copy number with gene expression levels. Proceeding under the hypothesis that cancer focal gains and losses are the result of selective pressure based on their regulatory effect on gene expression, we correlate the results of focal DNA copy number change, and gene expression to identify putative tumor suppressors.

## Materials and Methods

### DNA Isolation

DNA was isolated as described previously [22]. In brief, genomic DNA was purified from cells using the QIAamp DNA Blood Cell Culture Maxi Kit, (Qiagen Inc., Valencia, CA) according to manufacturer’s instructions. Quality was assessed by optical density 260/280 ratio using a spectrophotometer (Beckman-Coulter, Fullerton, CA) and by 0.8% agarose (SeaKem GTG, FMC BioProducts, Rockland, ME) gel electrophoresis in 1x TAE (Roche, Indianapolis, IN).

### DNA Copy Number in the NCI-60 Using four Microarray Platforms

DNA copy numbers for all genes were determined by the integration of probes from i) the Human Genome CGH Microarray 44A (Agilent Technologies, Inc., GEO accession GPL11068) with 44 k probes, ii) the H19 CGH 385K WG Tiling v2.0 array (Roche NimbleGen Systems, Inc., GEO accession GPL13786,), with 385 k probes, iii) the GeneChip Human Mapping 500 k Array Set (Affymetrix Technologies, Inc., GEO accession GPL3812) with 500 k probes, and iv) the Human Human1 Mv1_C Beadchip array (Illumina, GPL6983) with 1,100 k probes. Data for these microarrays can be accessed at CellMiner [21]. In addition, raw data has been deposited in the Gene Expression Omnibus (GEO) under the following accession numbers Agilent 44 k (GSE48568) Affymetrix 500 k (GSE32264), NimbleGen 385 K (GSE30291), Illumina 1 M (GSE47620).

### Probe Mapping and Intensities

Probes for the Agilent, NimbleGen and Illumina arrays were re-mapped to the latest HG19 reference using BLAST+ (Version 2.2.25) [23]. For the Affymetrix array, we used the latest annotation downloaded from the Affymetrix NetAffx website [24]. For each platform, we averaged the replicate samples (if available, see Table S1). Probe intensities were determined following manufacturers recommendations as described previously for the Agilent [25], NimbleGen Roche [26], Affymetrix [27], and Illumina [28] microarrays.

For all platforms, the log probe intensities for each sample were normalized by mean-centering, prior to all subsequent analysis. The mean of the log probe intensities was subtracted from all probe intensities for that sample.

### Segmentation of Regions with Consistent Copy Number

Segmentation refers to the partitioning of each chromosome into contiguous segments such that the copy number is the same within a segment and there is a significant difference in the copy number between adjacent segments. In our analysis, we used Circular Binary Segmentation (CBS) [29]. CBS returns the average probe intensity within each segment as an estimate of the log_2_ of copy number within that segment. Thus a mean probe intensity value of zero would correspond to a measured copy number of 2N (i.e. diploid), a value of -1 corresponds to copy number 1N and 1 corresponds to 4N.

Note that the Affymetrix 500 k data have been used before to detect regions of LOH (Loss of heterozygosity), however the algorithm used to detect the copy number variations was *pennCNV* which is unsuitable for genome-wide copy number estimation for cancer samples [30]. We have, therefore, re-analyzed the data using Circular Binary Segmentation (CBS).

### Combination of Copy Number Estimates from Four Platforms

We used a novel algorithm to combine the segmented copy number estimates from the four platforms for each cell line. We used the segmentation of the copy number to define *breakpoints* at the junction of two contiguous segments. At a breakpoint, a discrete jump (increase or decrease) of copy number occurs. These points correspond with locations of chromosomal breaks.

We align the breakpoints from the four platforms for the same cell line using the following method: Breakpoints from different platforms that are within 100,000 base pairs from each other and have the same direction of copy number change are matched with each other. This groups together breakpoints from different platforms that putatively refer to the same chromosomal break. Breakpoints that are not matched with any breakpoint from another platform are discarded. Then we compute an average breakpoint location from each group of matched breakpoints as the average of the locations of the breakpoints from the different platform. We compute the *average segment copy number* by averaging the segmented values between two adjacent averaged breakpoints over the four platforms.

For each gene, we find the segment in which it lies. The copy number for the gene is the *average segment copy number* for that segment. This assigns copy number estimates to 41 or more cell lines for 23,413 genes.

The copy number estimates for the genes were compared to copy number estimates from the Cancer Cell Line Encyclopedia (CCLE) [13] using 44 cell lines common to both datasets. We computed the Pearson correlation between our measurement of copy number and the CCLE copy number across the 44 cell lines for each gene.

### Prominent and Focal Gains and Losses

To identify the regions with the largest, most visually striking gains and losses, we set an arbitrary threshold of 1.5 on the absolute log_2_ copy number and joined segments that were less than 500 kilobases away from each other (including any segments between them).

For a systematic identification of all focal copy number gains (or losses) for each sample, we used the CBS (segmented) data to find portions of the genome that are higher (or lower) than both their left and right-hand neighbors. We used three criteria for calling a gain or loss focal: i) the segment must have a difference in log_2_ copy number of at least 0.3 from both its left and right-hand neighbors, both differences being either positive or negative; ii) the width of the segment must be less than 5 Mb; and iii) there should be more than 10 probes mapping within the segment. Any gene that has (partial or total) overlap with the segment is called focally gained or lost.

### Genomic Instability Parameters

Using the segmented copy number data, we calculated two forms of genomic instability; i) the proportion of the genome that has been gained or lost and, ii) the number of gains and losses. The proportion of the genome that is gained or lost was calculated based on the segmented values of the array CGH. We estimated this by taking the proportion of the probes falling within segments with absolute average intensities greater than 0.3 (a DNA copy number gain or loss of 0.46). The number of gains and losses was calculated as the total number (of gain/loss regions) with absolute average intensities greater than 0.3 with more than 10 probes mapping to the region.

### Gene Expression Determination and its Correlation to DNA Copy Number

Expression for 26,065 genes was taken as an integrated z-score of measurements from five gene expression platforms, as described previously [31]. Genes with expression z-scores were matched to genes with copy number. This resulted in 18,504 genes with both expression and copy number estimates. Copy numbers for these 18,504 genes were compared to gene expression using Pearson’s correlation (Table S3). The histogram of these correlations was plotted using *R* (version 2.15.2). The median correlations for all the genes, as well as for sets of known oncogenes and tumor suppressors, were calculated.

### Assessment of known and Putative Tumor Suppressors

We selected genes based on their meeting four criteria; i) statistically significant correlation between copy number and expression (False Discovery Rate FDR <0.05), ii) the gene being focally gained or lost in at least 3 samples (focal gains and losses as defined in the Segmentation section), iii) the number of cell lines with focal losses is at least 3 times greater than the number of cell lines with focal gains, iv) the genes were more than 2 million base pairs distance away from known tumor suppressors. Criterion 4 was used to remove “passenger” genes whose selection might be due to genomic proximity.

## Results

### The Array CGH Data can be Accessed and Visualized Using the CellMiner “Gene DNA Copy Number” web Analysis Tool

**Figure 1:**
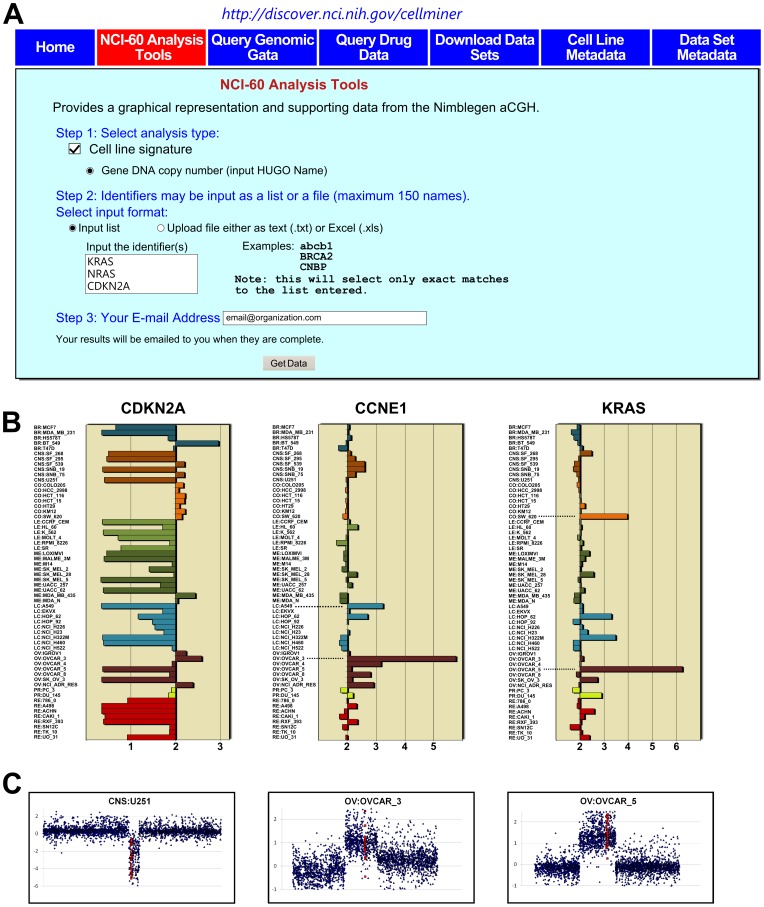
Web-based tool for accessing the four-platform aCGH data. **A.** The tool can be accessed at the CellMiner website by clicking on the “NCI-60 Analysis tools” tab (boxed in red). In this example, 3 cancer-associated genes are queried simultaneously: CDKN2A, CCNE1 and KRAS. **B.** The output includes a bar plot of the estimated copy number for each cell line. The x-axis is the DNA copy number. The y-axis shows the cell lines, with the bars colored based on tissue of origin. Bars to the left of 2N indicate loss whereas bars to the right indicate genomic gain. Dotted lines indicate cell lines with copy number gains in CCNE1 and KRAS **C.** A scatter plot is also provided for each cell line. The x-axis shows the chromosomal location. The y-axis shows the log2 intensity values on the left. The red dots indicate probes that fall within the gene. The blue dots indicate the flanking regions. The data are received as Excel files. See text for details.

To facilitate mining of the NCI-60 DNA copy number data, we introduce an intuitive tool to query and visualize the dataset. This tool is available at our CellMiner web site [21] within the “NCI-60 Analysis Tools” tab (Figure 1A). As shown in Figure 1A, users first select “Cell line signature” in Step 1, and then “Gene DNA copy number”. In Step 2, up to 150 genes of interest may be input by either typing in the gene names in the “Input the identifier” box, or uploading them as a text or Excel file using the “Upload file” radio button. In Step 3, users enter their e-mail address, and click “Get data”. Results will be sent by e-mail for each gene, with a link to download an Excel file. This file contains four worksheets: i) “DNA copy number” containing tabular mean intensity ratios (of the test DNA compared to presumed normal) and estimated DNA copy numbers, and a bar plot of the estimated DNA copy numbers (Figure 1B), ii) “Graphical Output” containing scatter-plots of the individual probe intensities for the gene of interest as well as 2MB flanking region for each cell line (Figure 1C), iii) “input” containing the normalized data for those probes that fall within a gene of interest (highlighted in yellow) as well as 2×10^6^ nucleotides of flanking region on each end, and iv) “Footnotes”. Figure 1 shows an example of 3 cancer-relevant genes (Figure 1A), CDKN2A encoding the Cyclin-Dependent Kinase Inhibitor 2A (p16^INK4a^, p19^ARF^), which is commonly deleted in cancers, CCNE1 encoding Cyclin E, which is commonly amplified in cancers, and KRAS encoding Kirsten Rat Sarcoma Viral Oncogene, which is activated in cancers by mutations and more rarely amplification. Panels B and C (Figure 1) show that many cell lines exhibit depletion of the CDKN2A locus (left panels), while ovarian cancer cells OVCAR3 and OVCAR5 show focal amplification of CCNE1 and KRAS, respectively.

**Figure 2:**
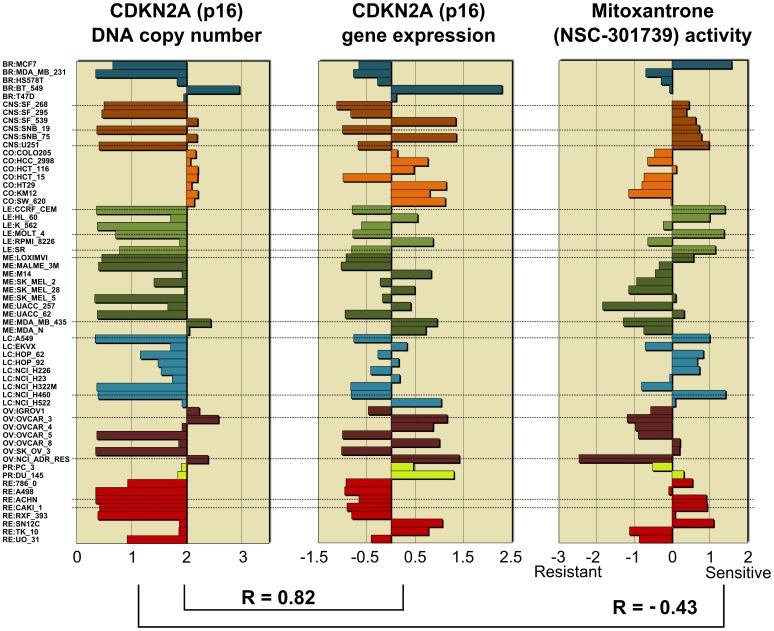
Example of integrated analysis using CellMiner. The leftmost plot shows a barplot of copy number values for CDKN2A obtained by querying CellMiner. The middle plot shows the gene expression and the rightmost plot shows the response to a Mitoxantrone, a drug with significant negative correlation with the copy number status of CDKN2A. Dotted lines indicate some of the cell lines where the direction of copy number alteration is in the same direction as the gene expression and in the opposite direction as the drug activity.

A unique feature of the CellMiner website is that the copy number pattern obtained from CellMiner for a gene can be used as input for the Pattern Comparison tool to find correlated genes expression and drug activity. Figure 2 shows the copy number for CDKN2A (p16), the gene with the highest-correlated expression (CDKN2A), and the drug whose response is the most negatively correlated (NSC-301739). The robust correlation between DNA copy number and transcript expression identify the robust affect that DNA copy number alteration has on transcript expression in this gene. The negative correlation of the DNA copy number to the drug activity identifies the FDA-approved drug mitoxantrone (NSC-301739) as being more active in multiple instances of cancer cells with CDKN2A deletion (Figure 2, right panel and dotted lines).

### Correlation with the Cancer Cell Line Encyclopedia

There are 44 cell lines common between the NCI-60 and the CCLE. Notably, the combined copy number estimates in the NCI-60 correlate well with the copy number estimates in the CCLE with a median correlation of 0.833. This is higher than the correlation to copy numbers from any individual platform (Agilent: Agilent: 0.660, NimbleGen: 0.448, Affymetrix: 0.821, Illumina: 0.804) implying that combining the platforms together improves the estimation. The higher correlation with the Affymetrix platform could be due to the fact the CCLE data was also generated on Affymetrix arrays (Affymetrix SNP 6.0).

### Widespread Alterations in DNA Copy Composition Occurs in the NCI-60 Cell Lines

**Figure 3:**
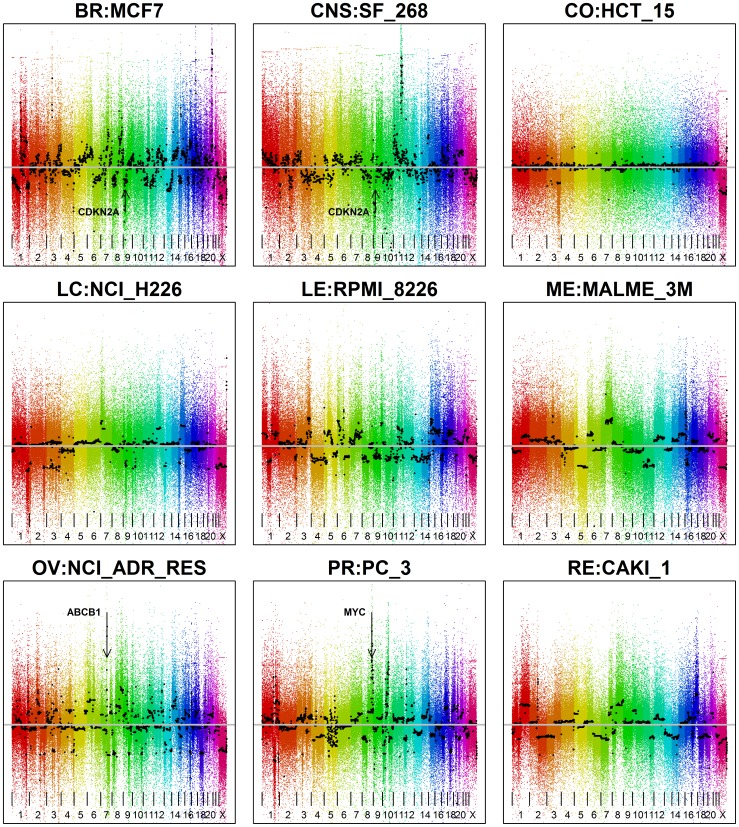
Whole genome visualization of aCGH results for the NCI-60. The x-axis is the chromosomal location of the probes, colored by chromosome number and ordered by genomic position. The y-axis is the log ratio of the probe intensities. The black horizontal marks indicate the average log_2_ copy numbers in each segment, as calculated by Circular Binary Segmentation (see Materials and Methods). The amount of scatter above and below the segments’ black marks indicates the level of probe variability. The locations of some cancer-related genes that have focal gains or losses are also indicated. High-resolution images for all the NCI-60 cell lines are available in Figure S1 and at our Website [21].

A global view of the NCI-60 genomic composition was generated using the CBS segmented aCGH results. Figure 3 displays representative examples of several genome variation types. The complete version for the NCI-60 is available in Figure S1 and at our website [21]. These displays reveal that most cell lines exhibit genomic alterations, including frequent genomic losses and gains, as well as altered ploidy. The types of variation in the genomes, however, vary widely within the NCI-60. Only some cell lines show normal (2N) copy number with few altered segments such as CO:HCT_15. Some have multiple altered genomic segments with approximately 2N overall copy number (e.g. RE:CAKI_1). Yet others have many altered segments in addition to being shifted from 2N, including BR:MCF7, CNS:SF_268, LE:RPMI_8226, ME:MALME_3M, OV:NCI_ADR_RES, and PR:PC_3. The data demonstrate the marked variability found in the abnormalities of the NCI-60 genomes.

The high intensity (absolute log_2_ values greater than 1.5, i.e. DNA copy numbers greater than 5.60 or less than 0.71) amplifications (gains) and deletions (losses), visualized in Figure 3 and Figure S1, are listed with their locations in Table S2 by cell line, due to their prospective importance. These large gains and losses have chromosome biases, with three chromosomes (9, 3 and 6) having multiple alterations in multiple cell lines, and one (chromosome 21) with no marked gains or losses. These data identify chromosome- and cell-specific focal amplifications and deletions.

### Global DNA Copy Number Alteration in the NCI-60

To further categorize the genomic copy number variations across the NCI-60, two parameters were derived from the aCGH data (Table 1). The “proportion of genome gained or lost” is the overall fraction of the genome that is gained or lost (compared to 2N); the “number of gained or lost regions” per genome represents the cumulative number of altered segments (gained or lost compared to 2N).

**Table 1:** Two measures of the genomic instability of the cell lines^a^.

Cell line	Proportion of genome gained/lost b			Number of gain/loss regions c		
	Gain	Loss	Total	Gain	Loss	Total
BR:MCF7	0.14	0.16	0.30	277	212	489
BR:MDA_MB_231	0.09	0.04	0.13	69	25	94
BR:HS578T	0.12	0.03	0.16	92	27	119
BR:BT_549	0.08	0.18	0.26	89	112	201
BR:T47D	0.25	0.06	0.31	146	34	180
CNS:SF_268	0.08	0.09	0.16	135	101	236
CNS:SF_295	0.07	0.11	0.19	64	72	136
CNS:SF_539	0.13	0.14	0.27	120	121	241
CNS:SNB_19	0.03	0.05	0.08	35	34	69
CNS:SNB_75	0.05	0.01	0.06	19	12	31
CNS:U251	0.05	0.06	0.11	50	41	91
CO:COLO205	0.08	0.13	0.21	54	82	136
CO:HCC_2998	0.01	0.00	0.01	4	4	8
CO:HCT_116	0.06	0.04	0.10	45	58	103
CO:HCT_15	0.00	0.05	0.05	14	30	44
CO:HT29	0.11	0.16	0.27	34	41	75
CO:KM12	0.05	0.10	0.15	49	51	100
CO:SW_620	0.05	0.04	0.09	46	37	83
LE:CCRF_CEM	0.03	0.02	0.05	40	38	78
LE:HL_60	0.07	0.12	0.18	89	114	203
LE:K_562	0.09	0.14	0.23	113	114	227
LE:MOLT_4	0.04	0.04	0.08	37	32	69
LE:RPMI_8226	0.12	0.14	0.26	133	80	213
LE:SR	0.03	0.04	0.08	20	22	42
ME:LOXIMVI	0.01	0.12	0.14	22	96	118
ME:MALME_3M	0.04	0.14	0.18	50	61	111
ME:M14	0.05	0.04	0.09	35	14	49
ME:SK_MEL_2	0.07	0.11	0.19	85	47	132
ME:SK_MEL_28	0.13	0.08	0.21	165	60	225
ME:SK_MEL_5	0.12	0.08	0.20	111	33	144
ME:UACC_257	0.09	0.21	0.29	89	124	213
ME:UACC_62	0.03	0.17	0.20	23	43	66
ME:MDA_MB_435	0.09	0.03	0.12	130	42	172
ME:MDA_N	0.09	0.00	0.09	64	11	75
LC:A549	0.11	0.08	0.19	113	91	204
LC:EKVX	0.13	0.03	0.16	124	34	158
LC:HOP_62	0.04	0.08	0.12	39	31	70
LC:HOP_92	0.14	0.06	0.19	97	51	148
LC:NCI_H226	0.06	0.12	0.18	23	85	108
LC:NCI_H23	0.06	0.02	0.09	61	22	83
LC:NCI_H322M	0.14	0.11	0.25	113	45	158
LC:NCI_H460	0.05	0.01	0.06	42	22	64
LC:NCI_H522	0.06	0.03	0.08	63	27	90
OV:IGROV1	0.00	0.01	0.01	2	31	33
OV:OVCAR_3	0.09	0.14	0.23	176	137	313
OV:OVCAR_4	0.14	0.14	0.28	152	112	264
OV:OVCAR_5	0.13	0.09	0.22	102	58	160
OV:OVCAR_8	0.13	0.09	0.21	117	77	194
OV:SK_OV_3	0.02	0.11	0.13	89	81	170
OV:NCI_ADR_RES	0.16	0.14	0.30	158	110	268
PR:PC_3	0.07	0.11	0.18	142	128	270
PR:DU_145	0.06	0.05	0.11	55	15	70
RE:786_0	0.05	0.12	0.17	46	54	100
RE:A498	0.08	0.23	0.31	72	156	228
RE:ACHN	0.08	0.04	0.12	80	27	107
RE:CAKI_1	0.11	0.09	0.19	65	45	110
RE:RXF_393	0.11	0.13	0.24	128	160	288
RE:SN12C	0.15	0.12	0.27	183	134	317
RE:TK_10	0.07	0.09	0.16	68	74	142
RE:UO_31	0.09	0.05	0.14	79	40	119

a The proportions and numbers of gained or lost segments are both based on the CBS segmented copy number analysis.

b The proportion of genome gained (or lost) is calculated as the fraction of genomic base-pairs with log2 gains (or losses) greater than 0.3 (or less than −0.3).

c The number of gained (or lost) regions is calculated as the number of contiguous genomic regions with log2 gains (or losses) greater than 0.3 (or less than −0.3).

Comparison of the two parameters (proportion and number of gains and losses) showed a highly statistically significant positive correlation (Pearson’s r = 0.76, p-value = 1.2×10^−12^), associating frequency to cumulative fraction of genomic alterations. The cell lines with the least frequent genomic alterations according to the first measure (proportion of genome gained or lost) are CO:HCC_2998 and OV:IGROV1, and those with the most are RE:A498 and BR:T47D. For the second measure (number of regions with gains/losses), the cells with the least alterations are CO:HCC_2998 and CNS:SNB_75, and the cell lines with the most alterations are BR:MCF7 and RE:SN12C.

### Prominent Areas of the Genome with Focal Copy Number Changes, and Their Relationship to Known and Prospective Tumor Suppressors

Next we searched for genomic copy number changes that were “focal” in nature. Our approach was to look for genomic segments with: i) a difference in log_2_ copy number of at least 0.3 from both its left and right-hand neighbors (the differences being either both positive or both negative); ii) a width less than 5 Mb; and iii) a minimum of 10 (aCGH) probes. Table 2 summarizes these focal alterations for known oncogenes and tumor suppressors. Table S3 provides the focal alteration status for all (18,504) genes with both copy number and gene expression (see column S), and their genomic positions (columns Q and R).

**Table 2:** Focal alterations and correlation between copy number alteration and expression for known tumor suppressors.

Gene	Cytoband	Entrez ID	Pearson correlation - copy number vs. expression			Number of cell lines with focal alterations	
			Correlation	P-value	FDR	Gains	Losses
EP300	22q13.2	2033	0.8266	0.0000	0.0000	0	0
CDKN2A	9p21.3	1029	0.8231	0.0000	0.0000	0	24
KDM5C	Xp11.22	8242	0.7974	0.0000	0.0000	0	0
PTEN	10q23.31	5728	0.7747	0.0000	0.0000	1	4
SMAD4	18q21.2	4089	0.7398	0.0000	0.0000	2	0
CIC	19q13.2	23152	0.7386	0.0000	0.0000	0	1
BRCA2	13q13.1	675	0.7171	0.0000	0.0000	0	0
MYCBP2	13q22.3	23077	0.7158	0.0000	0.0000	0	0
MDM2	12q15	4193	0.7147	0.0000	0.0000	1	0
SMARCB1	22q11.23	6598	0.7144	0.0000	0.0000	1	1
BAP1	3p21.1	8314	0.7138	0.0000	0.0000	0	1
NUP98	11p15.4	4928	0.7077	0.0000	0.0000	1	0
MLL3	7q36.1	58508	0.7012	0.0000	0.0000	0	1
E2F3	6p22.3	1871	0.6980	0.0000	0.0000	0	0
MAP2K4	17p12	6416	0.6916	0.0000	0.0000	0	1
CDK12	17q12	51755	0.6893	0.0000	0.0000	0	0
SETD2	3p21.31	29072	0.6780	0.0000	0.0000	1	0
CDC73	1q31.2	79577	0.6684	0.0000	0.0001	0	0
DNM2	19p13.2	1785	0.6668	0.0000	0.0001	0	0
TTK	6q14.1	7272	0.6662	0.0000	0.0001	0	0
MLH1	3p22.2	4292	0.6577	0.0000	0.0002	0	1
CD58	1p13.1	965	0.6540	0.0000	0.0002	1	0
MEN1	11q13.1	4221	0.6498	0.0000	0.0003	1	0
CTNNA1	5q31.2	1495	0.6295	0.0000	0.0012	0	1
BIRC6	2p22.3	57448	0.6208	0.0000	0.0020	1	0
XIST	Xq13.2	7503	0.6159	0.0000	0.0027	0	0
STAG2	Xq25	10735	0.6132	0.0000	0.0031	0	1
ATM	11q22.3	472	0.6106	0.0000	0.0036	0	0
PBRM1	3p21.1	55193	0.6087	0.0000	0.0040	0	1
ROBO1	3p12.3∶3p12.2	6091	0.5945	0.0000	0.0088	0	1
FBXW7	4q31.3	55294	0.5845	0.0000	0.0149	0	2
UBR4	1p36.13	23352	0.5844	0.0000	0.0185	0	0
E2F1	20q11.22	1869	0.5754	0.0000	0.0237	1	0
LIG3	17q12	3980	0.5688	0.0000	0.0329	0	1
SMARCA4	19p13.2	6597	0.5586	0.0000	0.0651	0	0
TP53	17p13.1	7157	0.5536	0.0000	0.0684	0	2
AMER1	Xq11.2	139285	0.5399	0.0000	0.1519	0	0
TSC2	16p13.3	7249	0.5382	0.0000	0.1373	1	0
NF1	17q11.2	4763	0.5094	0.0000	0.4644	1	0
ING4	12p13.31	51147	0.5072	0.0000	0.5063	0	0
ARID1A	1p36.11	8289	0.4958	0.0001	0.7943	0	0
CDKN2C	1p32.3	1031	0.4790	0.0001	1.0000	0	3
ATRX	Xq21.1	546	0.4643	0.0002	1.0000	0	0
BRCA1	17q21.31	672	0.4582	0.0002	1.0000	0	0
TSC1	9q34.13	7248	0.4553	0.0003	1.0000	0	0
NOTCH2	1p12∶1p11.2	4853	0.4439	0.0004	1.0000	0	0
EZH2	7q36.1	2146	0.4433	0.0004	1.0000	0	0
B2M	15q21.1	567	0.4382	0.0005	1.0000	1	1
ADAMTS18	16q23.1	170692	0.4238	0.0007	1.0000	0	0
STK11	19p13.3	6794	0.4129	0.0012	1.0000	0	1
VHL	3p25.3	7428	0.4078	0.0012	1.0000	0	0
MGA	15q15.1	23269	0.4055	0.0013	1.0000	0	2
MSH2	2p21	4436	0.4039	0.0014	1.0000	0	0
NPM1	5q35.1	4869	0.3998	0.0017	1.0000	0	0
CYLD	16q12.1	1540	0.3977	0.0017	1.0000	1	0
ASXL1	20q11.21	171023	0.3950	0.0018	1.0000	0	0
ACVR2A	2q22.3	92	0.3835	0.0025	1.0000	2	1
CREBBP	16p13.3	1387	0.3744	0.0032	1.0000	0	1
TET2	4q24	54790	0.3607	0.0046	1.0000	1	0
KDM6A	Xp11.3	7403	0.3605	0.0047	1.0000	0	0
MSH6	2p16.3	2956	0.3583	0.0049	1.0000	0	0
JAK1	1p31.3	3716	0.3553	0.0053	1.0000	1	0
RB1	13q14.2	5925	0.3520	0.0058	1.0000	0	0
APC	5q22.2	324	0.3433	0.0072	1.0000	0	0
GATA3	10p14	2625	0.3332	0.0093	1.0000	0	0
PIK3R1	5q13.1	5295	0.3029	0.0186	1.0000	0	0
NOTCH1	9q34.3	4851	0.2858	0.0269	1.0000	0	0
WT1	11p13	7490	0.2734	0.0362	1.0000	1	1
NKX3-1	8p21.2	4824	0.2691	0.0376	1.0000	0	0
E2F2	1p36.12	1870	0.2661	0.0399	1.0000	0	0
DNMT3A	2p23.3	1788	0.2587	0.0459	1.0000	0	0
NF2	22q12.2	4771	0.2532	0.0510	1.0000	1	1
BCOR	Xp11.4	54880	0.2451	0.0591	1.0000	0	0
FUBP1	1p31.1	8880	0.2347	0.0711	1.0000	0	0
TGFB1	19q13.2	7040	0.2171	0.1017	1.0000	0	0
CEBPA	19q13.11	1050	0.2138	0.1040	1.0000	0	0
PHOX2B	4p13	8929	0.2104	0.1097	1.0000	0	1
PTCH1	9q22.32	5727	0.1945	0.1364	1.0000	0	0
TAF1L	9p21.1	138474	0.1941	0.1372	1.0000	0	0
TUSC3	8p22	7991	0.1669	0.2025	1.0000	1	0
F8	Xq28	2157	0.1621	0.2201	1.0000	0	0
PRDM1	6q21	639	0.1549	0.2374	1.0000	0	0
HNF1A	12q24.31	6927	0.1156	0.3792	1.0000	1	0
TGFBR2	3p24.1	7048	0.1134	0.3883	1.0000	0	1
RUNX1	21q22.12	861	0.1109	0.3987	1.0000	0	0
TEK	9p21.2	7010	0.0945	0.4725	1.0000	2	0
GATA1	Xp11.23	2623	0.0857	0.5189	1.0000	0	0
MYH2	17p13.1	4620	0.0851	0.5218	1.0000	0	1
ABL2	1q25.2	27	0.0824	0.5312	1.0000	0	0
SI	3q26.1	6476	0.0736	0.5797	1.0000	0	1
TUSC1	9p21.2	286319	0.0693	0.6019	1.0000	0	0
SOCS1	16p13.13	8651	0.0648	0.6229	1.0000	0	0
TNFAIP3	6q23.3	7128	0.0423	0.7484	1.0000	0	0
CDH1	16q22.1	999	0.0416	0.7525	1.0000	0	0
MAST4	5q12.3	375449	0.0265	0.8410	1.0000	0	1
PAX5	9p13.2	5079	0.0004	0.9973	1.0000	0	0
DCC	18q21.2	1630	−0.0521	0.6952	1.0000	0	2
IRF6	1q32.2	3664	−0.0540	0.6819	1.0000	0	0
WNK2	9q22.31	65268	−0.1339	0.3078	1.0000	0	1
VHLL	1q22	391104	−0.1505	0.2596	1.0000	0	0
NOTCH3	19p13.12	4854	−0.2952	0.0232	1.0000	0	0

**Figure 4:**
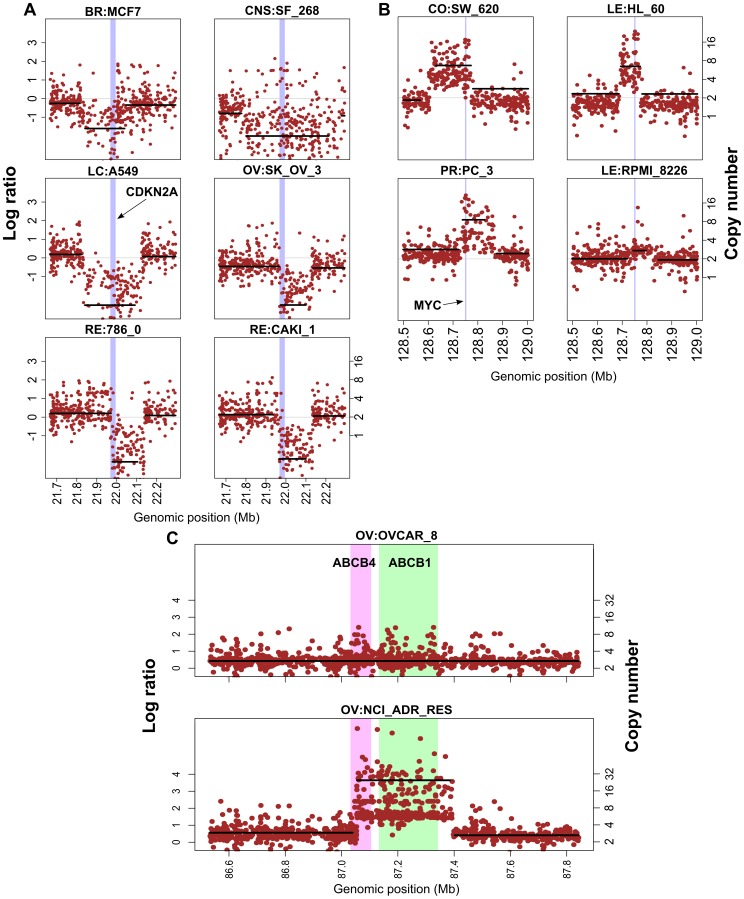
Plots of probe intensities and segmented averages for cancer interesting genes. **A.** CDKN2A and flanking sequence on chromosome nine for six cell lines. The central vertical lilac region delineates the gene location. **B**. MYC and flanking sequence on chromosome eight for five cell lines. The central vertical lilac region delineates the gene location. **C**. ABCB1 (MDR1), ABCB4 and flanking sequence on chromosome 7 for the parental OVCAR_8 and its drug-resistant derivative NCI_ADR_RES. The green and pink central vertical regions delineate the locus of ABCB1 and ABCC4, respectively. In A, B, and C the x-axis is the nucleotide location. The y-axis values on the left are the average log intensity ratios, and on the right are estimated DNA copy numbers. The black horizontal lines show the average log intensity ratio in each segment while the brown points show the log intensity ratios for each probe.

The most commonly focally deleted segment occurs in 24 cell lines, and contains the CDKN2A tumor suppressor gene (p16^INK4a^ and p14^ARF)^ on chromosome 9 (Figure 1B, 2 and 4A). The CDKN2A deletions occur in most of the NCI-60 tissue types, with the highest incidence in renal (6 out of 8 lines) and CNS cells (4 out of 6 lines). CDKN2A deletions are less frequent in breast (1 out of 5) and ovarian (2 out of 7) and absent in the colon and prostate lines. The detailed data for CDKN2A is found in Table S3 (column Q). The next most commonly deleted tumor suppressor gene is PTEN on chromosome 10 (Table 2 and Table S3), which is markedly under-represented in 4 cell lines: CNS:SF_539, LE:CCRF_CEM, PR:PC_3 and RE:RXF_393. It is also focally gained in OV:OVCAR_4. Notably TP53, which is inactivated by mutations in 47 of the NCI-60 [3], [32] (our submitted results) has focal loss in only two cell lines LE:HL_60, RE:TK_10 (Table S3), demonstrating specificity in mechanism of function knockdown of tumor suppressors.

For the known oncogenes, the most frequent focal gain occurs in the CCND1 (cyclin D1) gene on chromosome 11, and in MYC, on chromosome 8. CCND1 has focal gains in 4 cell lines (CNS:SF_295, ME:SK_MEL_28, ME:SK_MEL_5, RE:TK_10) including 2 melanomas. MYC is amplified in four cell lines CO:SW_620, LE:HL_60, LE:RPMI_8226 and PR:PC_3 (Figure 4B).

Besides known oncogenes and tumor suppressors, one of the most intense amplifications was found in the OV:NCI_ADR_RES cell line on chromosome 7q21.12 (Figure 3, lower left panel and Figure 4C). This amplification encompasses two efflux pump ABC transporter genes, ABCB1 and ABCB4 (Figure 4C), and is consistent with the high doxorubicin (adriamycin) resistance of this cell line [33], [34]. Other than this chromosome 7 focal amplification, the OV:NCI_ADR_RES cell line shows an aCGH profile comparable to its parental line OV:OVCAR_8 (Figure S1).

### Correlation between Gene Expression and DNA Copy Number

**Figure 5:**
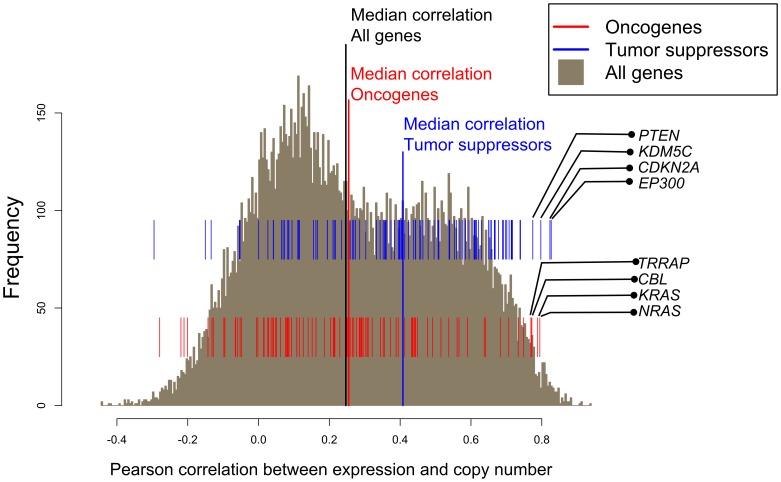
Correlation between DNA copy number alterations and transcript expression for all genes. Histogram of the Pearson’s correlations between copy number and gene expression for the complete set of 18,504 genes with both values available. The lower and upper sets of tick marks above the x-axis show the correlations for individual oncogenes (in red) and tumor-suppressors (in blue), respectively.

To determine the relationship between DNA copy number and transcript expression levels, we calculated the correlations between the two parameters for all (18,504) genes with both copy number and gene expression. Table 2 and Table S3 give these correlation values, as well as the corresponding p-value and FDR for the tumor suppressors, and all genes, respectively. The histogram in Figure 5 shows that the median Pearson’s correlation is r = 0.247, providing a global indicator of the influence of gene copy number on expression.

The median correlation of the combined data is higher than any individual platform (Agilent: 0.212, NimbleGen: 0.149, Affymetrix: 0.242, Illumina: 0.226), again implying that the combined data improves the copy number estimation over using any individual platform.

The subset of 101 known tumor suppressors had a significantly higher median correlation (r = 0.408, Figure 5) than the whole genome (r = 0.247, Figure 5). The subset of 96 known oncogenes showed only slightly higher correlation compared the overall genome (median r = 0.255; Figure 5). These results demonstrate that gene loss influences expression of known tumor suppressors to a greater degree than either the “all genes” or oncogenes groups.

### Identification of Novel Putative Tumor Suppressor Genes

Since focal changes in DNA copy number of known tumor suppressor genes (Figure 1B and C, Figure 3, Table 2) showed highly significant correlation to their transcript expression levels (Figure 5, Table 2), we used this characteristic to search for and identify additional genes with potential relation to cancer. Our approach was based on the results for the known tumor suppressors CDKN2A and PTEN (Table 3). The selection criteria for novel genes required: i) correlations between DNA copy number and transcript levels significant to a FDR of 0.05, ii) focal gains or losses in at-least three cell lines [focal changes were defined as gains or losses smaller than 5 Mb that overlap the gene], and iii) a 3∶1 or greater ratio for the number of cell lines with losses compared to gains. In addition, we required that the genes pass a fourth criteria that there should be no known tumor suppressors within 2 MB (to avoid detecting “neighbors” of known driver tumor suppressors).

**Table 3:** Selected known and putative tumor suppressors.

Gene	Cytoband	Pearson correlation			Number of cell lines with focal alterations		Known relation to cancer c
		Correlation	P-value	False detection ratio (FDR)	Gains	Losses	
CDKN2A a	9p21.3	0.8231	7.08E-16	1.31E-11	0	24	Known tumor suppressor
PTEN a	10q23.31	0.7747	3.82E-13	6.97E-09	1	4	Known tumor suppressor
RNF121 b	11q13.4	0.6284	7.62E-08	0.0013	1	3	In a region associated with breast cancer susceptibility (PMID:19205878)
IL18BP b	11q13.4	0.6047	3.10E-07	0.0050	1	3	In a region associated with breast cancer susceptibility (PMID:19205878)
NUMA1 b	11q13.4	0.7396	1.48E-11	2.67E-07	1	3	In a region associated with breast cancer susceptibility (PMID:19205878)
LAMTOR1 b	11q13.4	0.7376	1.79E-11	0.0000	1	3	Downregulation induces p53 dependant apoptosis (PMID:22513874)
NCOR1 b	17p12	0.7699	6.47E-13	1.18E-08	0	4	Upregulation correlates with better prognosis in breast cancer (PMID:16019133)
FLCN b	17p11.2	0.7087	2.38E-10	0.0000	0	3	A suspected tumor suppressor gene. Inactivating mutations in this gene causes Birt-Hogg-Dubé syndrome (PMID: 23223565)
PEMT b	17p11.2	0.7476	6.75E-12	1.22E-07	0	3	Lower expression in HCC compared to normal liver and low expression correlates with poorer survival (PMID:12931022). Higher expression induces apoptosis (PMID:11960751)
PTRH2 b	17q23.1	0.7750	3.67E-13	0.0000	1	3	Mediates apoptosis. Downregulation of expression increases metastasis (PMID:21886829)
SDF2L1 b	22q11.21	0.6655	6.60E-09	0.0001	0	3	Low expression correlated with poor survival in breast cancer (PMID:19513569)
DEPDC5 b	22q12.2	0.5891	7.38E-07	0.0117	0	3	Mutations associated with progression to hepatocellular carcinoma in chronic hepatitis C virus carriers (PMID:21725309)

a Two known tumor suppressors that have significant correlation between gene expression and copy number and an abundance of focal losses compared to gains.

b Ten out of 22 additional genes identified using the same criteria that are also not proximal (within 2 MB) to a known tumor suppressor (Complete list in Table S4).

c Column contains literature connections between the gene and cancer, along with the Pubmed IDs.

We assessed all 18,504 genes that have both gene expression and copy number estimates to identify those that passed the above criteria. Thirty one genes passed criteria 1–3 (Table S4), and 22 satisfied all four criteria (Indicated in column U and highlighted in green). Those genes group into 12 “gene clusters” such that genes in the same cluster are adjacent to each other and have copy numbers that are highly correlated (to each other) across the NCI-60 (Pearson correlation >0.8), indicating that they are largely lost or gained as a group. The 22 novel tumor suppressor clusters are at cytobands 11q13.4, 17p12, 17p11.2, 17q23.1, 21q11.2, 21q21.1, 22q11.21, 22q12.2, 22q13.1 and Xp22.31. Table 3 lists ten of the genes that fall within these clusters and have been reported to exhibit tumor suppressor characteristics.

## Discussion

In the current study we combined data on the NCI-60 cell line panel from four high-resolution array CGH platforms. Combining the four platforms yields a dataset with i) increased probe coverage, ii) higher correlation to the copy number estimates from the CCLE (Cancer Cell Line Encyclopedia), and iii) higher correlation to gene expression, indicating better estimates that any one platform alone.

The dataset adds to the array of molecular data available for the NCI-60, facilitating integrative (“integromic”) [4], [8], [32], [35] studies of cancer biology and molecular pharmacology. The data and analysis tools to facilitate its use are publicly available at our NIH CellMiner web suite [21] (Figure 1A). We also provide an example of the kind of integrative analysis that can be done. Comparing the DNA copy number for CDKN2A, a known tumor suppressor to its mRNA expression reveals the robust manner in which this molecular alteration is associated with the genes expression, and its frequent inactivation in the NCI-60 (see Figure 1 and Table S3). Comparing the DNA copy number for CDKN2A to the compound database reveals the FDA-approved drug mitoxantrone (NSC301739) as being more active in cell lines with CDKN2A knockout (Figure 2).

The patterns of gains and losses in the cell lines encompass a wide spectrum, with different patterns of variation likely representing differences in the molecular malfunctions within the cells (Figure 3, Figure S1 and website [21]). Using the identified areas of relative focal chromosomal gain or loss (size <5x10^6^ bp and amplitude >0.3 of the log_2_ of the copy number), we calculate two new measures of genomic instability, the proportion of the genome gained or lost, and the total number of gains and losses for a cell line (Table 1).

Between OV:OVCAR_8 and its adriamycin-resistant derivative OV:NCI_ADR_RES, we find a large number of copy number differences (15 focal gains and 5 losses in OVCAR_8 that are not present in NCI_ADR_RES and 20 focal gains and 9 losses in NCI_ADR_RES that are not present in OVCAR_8) (Figure 4C and Figure S1 and website [21]). The most striking is the small, focal (∼3×10^5^ nucleotides) high intensity amplification in NCI_ADR_RES (Figure 3 and Figure S1) that includes two efflux pump genes, ABCB1 (MDR1) and ABCB4 (Figure 4C). ABCB1 has previously been shown to be up-regulated in the NCI_ADR_RES [33] and other multiple-drug resistant cell lines [33], [34], [36]–[38]. Thus, our results confirm over-expression of ABCB1, and add up-regulation of ABCB4 in NCI_ADR_RES (as compared to OVCAR_8) and associate this increase to increased DNA copy number.

Our data demonstrates and catalogues prominent focal gains and losses of cancer-related genes in multiple cell lines (Table 2). Among the tumor suppressors, both the CDKN2A (p16) and PTEN losses are consistent with prior reports of deletion in cancers [39], [40]. The oncogene MYC, with focal gains in four cell lines (leukemia HL60 and RPMI_8226, colon carcinoma SW620 and prostate carcinoma DU_145; Table S3) has been reported to be amplified in prior reports [41].

The median positive correlation (r = 0.247) between DNA copy number and transcript expression (Figure 5) is consistent with prior results [2], [42]–[44]. Interestingly, we found a markedly higher correlation for the 101 known tumor suppressors (r = 0.408), than for the 96 oncogenes (r = 0.255). This implies loss of copy number is a stronger driver of altered gene expression for tumor suppressors than gain of copy number is for oncogenes. To the best of our knowledge, our study is the first attempt to compare focal DNA copy number and transcript expression changes across multiple cancer types.

The known tumor suppressors CDKN2A and PTEN provide criteria of recurrent focal DNA gains or losses that correlate well with transcript expression. They imply that DNA copy number correlated with expression level change is an indicator for cancer-relatedness in genes. Using these criteria to identify novel putative tumor suppressor genes (Table S4), we find 12 chromosomal segments containing 22 correlated genes (without nearby known oncogenes or tumor suppressors). Of these, five contain at least one gene with prior literature association with cancer (Table 3). The area with the most genes occurs in chromosome 17. It contains 10 genes with correlated expression level change, including four with prior association with cancer (NCOR1, FLCN, PEMT and PTRH2). Among these, FLCN has been suspected to be a tumor suppressor gene whose inactivation by mutations is causative of Birt-Hogg-Dubé syndrome, whose symptoms include susceptibility to renal cancers [45].

In summary, we present a novel combination of chromosomal segmentation results from multiple aCGH platforms and provide both an intuitive web-based public resource [21] and a high-resolution and improved quality view of the genome-wide copy number variation of the NCI-60 cancer cell lines. We identify a catalog of focal copy number gains and losses for both important known tumor suppressors and oncogenes, and novel tumor suppressor gene candidates. Copy number changes for any gene of interest can be interrogated using the web-based CellMiner tools [21], which enable users to connect the largest publicly available drug database with a full array of genomic databases.

## Supporting Information

Figure S1 Whole genome visualization of aCGH results for all cell lines from the NCI-60. The x-axis is the chromosomal location of the probes, colored by chromosome number and ordered by genomic position. The y-axis is the log ratio of the probe intensities, shown on the left side of the plots, and the estimated DNA copy number, shown on the right side of the plot. The black horizontal lines indicate the average log_2_ copy numbers in each segment, as calculated by CBS. The amount of scatter above and below the segments black lines indicate the level of probe variability. CO:HT29 has data only on the Agilent platform, which makes the number of plotted points much lower than the other cell lines.
(TIF)

Table S1 Details of the four aCGH platforms on which the NCI-60 data are available.
(XLSX)

Table S2 List of the highest intensity gain and loss regions in each chromosome for each cell line.
(XLSX)

Table S3 Correlation between gene expression and copy number for all genes along with the number of cell lines with focal gains or losses.
(XLSX)

Table S4 Genes that satisfy the three criteria (see article) for a putative new tumor suppressor.
(XLSX)
