# Supplementary figures and images for: High Resolution Copy Number Variation Data in the NCI-60 Cancer Cell Lines from Whole Genome Microarrays Accessible through CellMiner
Source: PLoS One. 2014 Mar 26;9(3):e92047. doi: 10.1371/journal.pone.0092047 (PMC3966786; doi:10.1371/journal.pone.0092047)

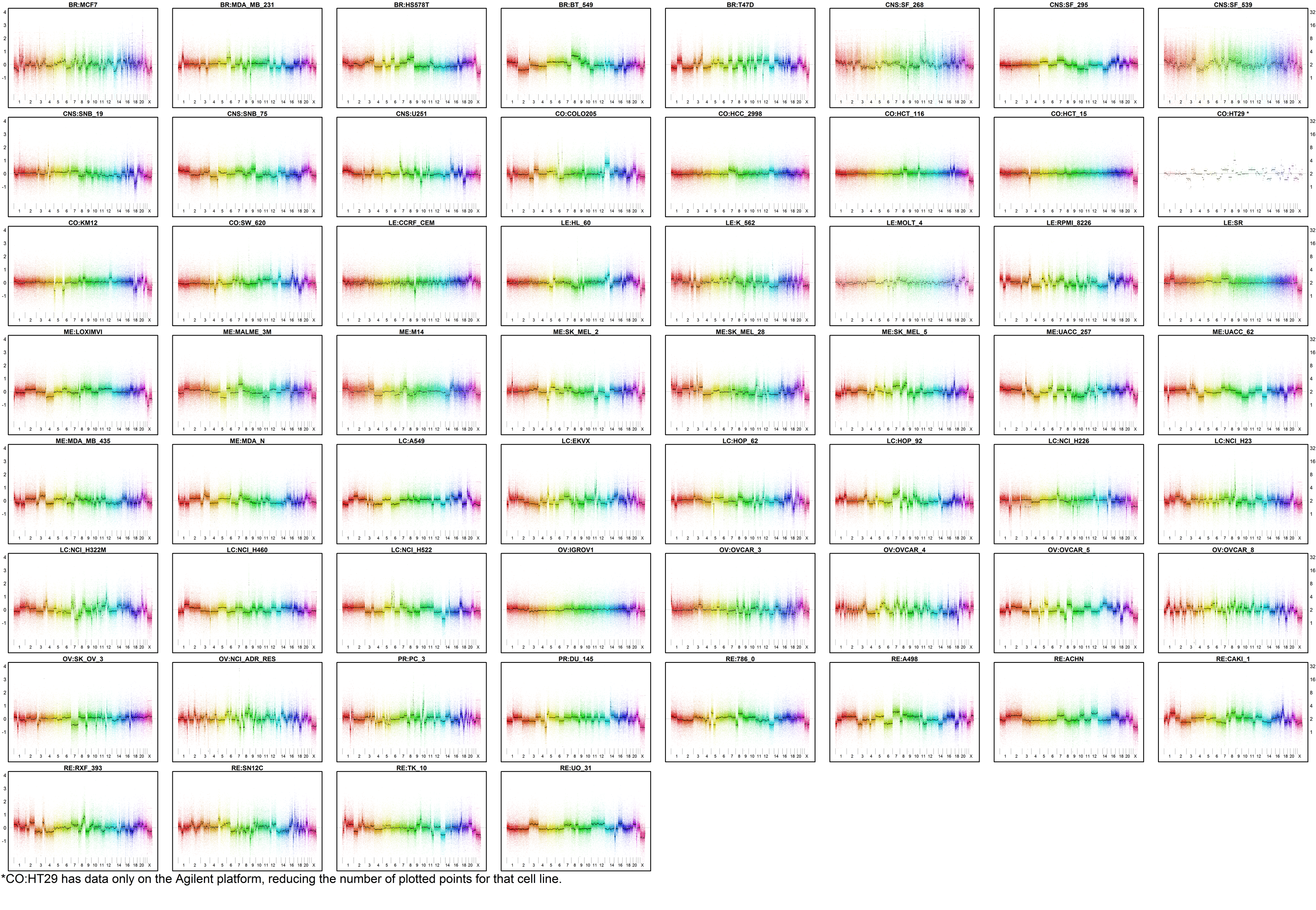

Supplement: Figure S1 — Whole genome visualization of aCGH results for all cell lines from the NCI-60. The x-axis is the chromosomal location of the probes, colored by chromosome number and ordered by genomic position. The y-axis is the log ratio of the probe intensities, shown on the left side of the plots, and the estimated DNA copy number, shown on the right side of the plot. The black horizontal lines indicate the average log2 copy numbers in each segment, as calculated by CBS. The amount of scatter above and below the segments black lines indicate the level of probe variability. CO:HT29 has data only on the Agilent platform, which makes the number of plotted points much lower than the other cell lines. (TIF) [file pone.0092047.s001.tif]
